# Pretreatment with probiotics ameliorate gut health and necrotic enteritis in broiler chickens, a substitute to antibiotics

**DOI:** 10.1186/s13568-020-01153-w

**Published:** 2020-12-17

**Authors:** Danish Sharafat Rajput, Dong Zeng, Abdul Khalique, Samia Sharafat Rajput, Hesong Wang, Ying Zhao, Ning Sun, Xueqin Ni

**Affiliations:** 1 Animal Microecology Institute, College of Veterinary, Sichuan Agricultural University, 611130 Chengdu, China; 2 Department of Gastroenterology, Nanfang Hospital, Southern Medical University, Guangdong, China; 3 Department of Veterinary Microbiology, Faculty of Animal Husbandry and Veterinary Sciences, Sindh Agriculture University, 70050 Tandojam, Pakistan

**Keywords:** *Clostridium perfringens*, Necrotic enteritis, Gut health, Probiotics, Antibiotics, Intestinal immunity, Broilers

## Abstract

Necrotic enteritis (NE) is being considered as one of the most important intestinal diseases in the recent poultry production systems, which causes huge economic losses globally. NE is caused by *Clostridium perfringens*, a pathogenic bacterium, and normal resident of the intestinal microflora of healthy broiler chickens. Gastrointestinal tract (GIT) of broiler chicken is considered as the most integral part of pathogen’s entrance, their production and disease prevention. Interaction between *C. perfringens* and other pathogens such as *Escherichia coli* and *Salmonella* present in the small intestine may contribute to the development of NE in broiler chickens. The antibiotic therapy was used to treat the NE; however European Union has imposed a strict ban due to the negative implications of drug resistance. Moreover, antibiotic growth promoters cause adverse effects on human health as results of withdrawal of antibiotic residues in the chicken meat. After restriction on use of antibiotics, numerous studies have been carried out to investigate the alternatives to antibiotics for controlling NE. Thus, possible alternatives to prevent NE are bio-therapeutic agents (Probiotics), prebiotics, organic acids and essential oils which help in nutrients digestion, immunity enhancement and overall broiler performance. Recently, probiotics are extensively used alternatives to antibiotics for improving host health status and making them efficient in production. The aim of review is to describe a replacement to antibiotics by using different microbial strains as probiotics such as bacteria and yeasts etc. having bacteriostatic properties which inhibit growth of pathogens and neutralize the toxins by different modes of action.

## Introduction

Necrotic enteritis (NE) is an extensive bacterial disease of broilers which causes significant economic loss by damaging the intestinal mucosa. It has been estimated that NE causes over US$ 6 billion economic losses every year globally (Moore 2016) and has become fourth leading bacterial induced food-borne disease in the United States (Flynn 2014). The economic loss of NE results in impaired growth performance, decreased weight gain and increased feed conversion ratio (FCR). Also, high mortality and greater medication cost make it one of the most costly diseases (Mot et al. 2014; Gaucher et al. 2015). Unfortunately, several predisposing factors such as high fish meal, protozoal infection and stress create promising environment for *C. perfringens* to proliferation (Rodgers et al. 2015). The gastro intestinal tract (GIT) is considered as the most integral part of productivity, pathogen entrance and disease prevention. The gut health depends upon nutritional and health status of poultry birds including immune system, balanced gut microflora and intestinal mucosa. The gut health affects not only digestion and absorption of nutrients but also fights against pathogens (Stanley et al. 2012; Bailey 2013). However, disturbance in any physiological process could lead to development of the disease (Dekich 1998) and may affect overall bird growth performance. Therefore, great attention is needed to the health of GIT because it is naturally protected by probiotic bacteria, present in the intestine. When probiotics fail to protect the host against harmful bacteria and viruses, the pathogens start invading tissues by producing lethal toxins and metabolites (Abaidullah et al. 2019). The composition of live microorganisms depend on age and geography; which change under the influence of various factors such as diet variety, the GIT transit time, and intestinal pH (Morgan et al. 2013a, b), leading to emergence of the disease.

Commonly, antibiotic growth promoters (AGPs) were used to treat the NE but use of AGPs have been restricted by European Union (EU) since 2006 (Caly et al. 2015; Lekshmi et al. 2017; Khalique et al. 2019). Moreover, the use of AGP was declared as an alarming threat to public health by WHO in 2012 (Organization 2012) and led to restriction of antimicrobial therapy. After ban on antibiotics, there is an urgent need to search effective alternative strategy to antibiotic growth promoters that could support health and growth performance of broilers. Subsequently, the focus of researchers on substitute strategies have been accelerated to secure livestock animals and poultry birds. Therefore, several amazing non-antibiotic therapies include the administration of competitive exclusion (CE) such as probiotics (Wang et al. 2017; Whelan et al. 2018), prebiotics (Keerqin et al. 2017) and essential oils (Brenes and Roura 2010) have shown bacteriostatic properties (Calik and Ergün 2015). Among non-antibiotic strategies, probiotics have been widely used as an alternative to antibiotics which increase the population of beneficial bacteria and promote intestinal health of the host (M’Sadeq et al. 2015). In addition, probiotic bacteria help in digestion and absorption of the nutrients by producing hydrolytic enzymes. Besides, supplementation of probiotics enhance immunity by modulating intestinal microbiota and reduce pathogenic colonization such as *C. perfringens* (Hofacre et al. 2019). Therefore, the aim of this review is to summarize the effectiveness of different probiotic strains against *C. perfringens* in broilers through different modes of action which could be used as alternative to antibiotics.

### ***Clostridium perfringens*** and toxinotypes

**Fig. 1:**
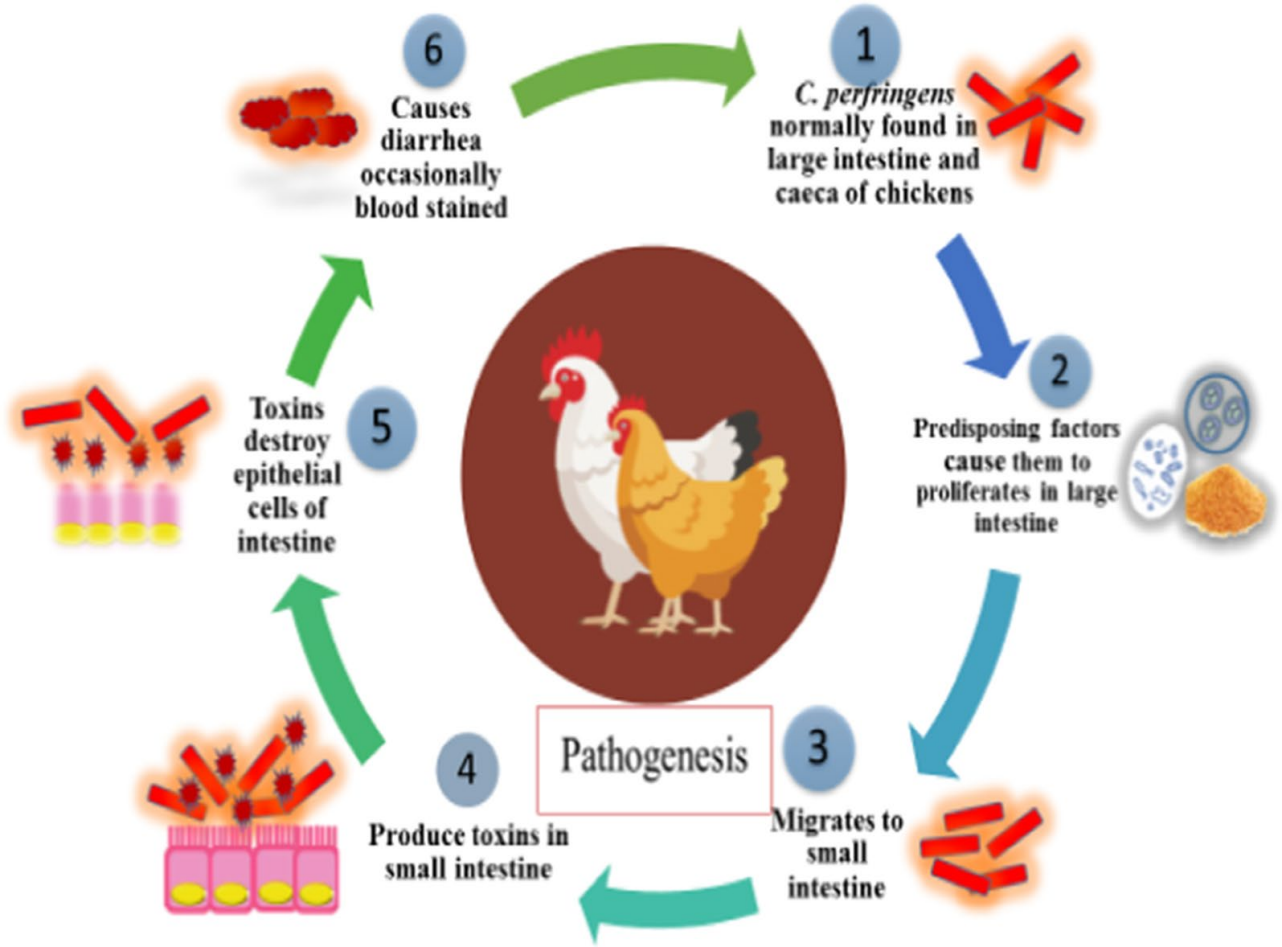
Pathogenesis of necrotic enteritis in broiler chickens causes destruction of epithelial cells of intestine that leads to blood-stained diarrhea

*Clostridium perfringens* (*C. perfringens*) is a gram-positive, rod shaped, spore-forming pathogenic bacterium which is believed to be the main pathogen of NE and subclinical necrotic enteritis (SNE) in poultry associated with chronic damage to the intestinal epithelium (Prescott et al. 2016; Bhogoju et al. 2018; Khalique et al. 2020). To-date, several types of *C. perfringens* have been isolated in different poultry species such as type A, B, C, D, E and G (Keyburn et al. 2010) and each type produces different toxins. In poultry, NE and SNE are mainly caused by type A and G which are normally found in the intestine of healthy poultry birds with less than 10² to 10^4^ colony-forming units (CFU) per gram of intestinal contents compared to 10^7^ − 10^9^ CFU/g in infected birds (Timbermont et al. 2009; Shojadoost et al. 2012). Several predisposing factors such as coccidiosis, dysbiosis and high fish meal increase the counts of *C. perfringens*. After exposure to predisposing factors, *C. Perfringens* starts secreting harmful substances which play an important role to the development of the disease. The harmful substance like perfrin inhibits the growth of useful bacteria by nutrients competition and creates dysbiosis in the gut (Timbermont et al. 2010). In addition to *C. perfrigens*, NE is also caused by toxins produced by *C. perfringens* type A and G which produce alpha (α) toxins and netB toxins respectively (Cooper and Songer 2009). For understanding the mechanism of disease, the pathogenesis of NE is shown in (Fig. 1). However, control of *C. perfringens* is ultimate control of harmful toxins which cause necrosis of intestinal mucosa and results in impaired absorption of nutrients.

Recently discovered toxin produced by *C. perfringens* is NetB toxin. NetB producing strains are capable to infect broiler chickens and lead to NE outbreaks (Wu et al. 2010). The toxinotype G strain produces NetB toxins (Rood et al. 2018) and has been recognized as pore-forming toxin (Savva et al. 2013; Yan et al. 2013) coded by plasmid genomes (Keyburn et al. 2008). The isolated strains of *C. perfringens* from healthy broilers do not carry NetB gene (Lacey et al. 2016) while infected birds usually have NetB gene (Keyburn et al. 2010). Thus, NetB gene and other genomic regions are well-known for causing NE in broilers (Parreira et al. 2017). The mechanism of *C. perfringens* virulence strains are still unknown (Lacey et al. 2016; Prescott et al. 2016). However, different *C. perfringens* strains possess different virulence levels. For measuring virulence level, two toxins i.e. NE18 and NE36 isolated from *C. perfringens* infected broilers which contains NetB gene (Lacey et al. 2018); comparatively NE36 shows greater virulence than NE18 (Keyburn et al. 2013). Additionally, *C. perfringens* having NetB gene can be the reason of causing NE in the absence of alpha α-toxins (Keyburn et al. 2010) (Table 1).

**Table 1:** Protozoans/CP and their dose which cause necrotic enteritis in broiler chickens

Serial no.	Pathogen/protozoans	Infection dose	References
1.	*Eimeria necatrix*	2 × 10^4^ oocysts/bird	De Cesare et al. (2020)
2.	*Eimeria. maxima*	2–5 × 10^4^ oocysts/bird	Gholamiandehkordi et al. (2007), Collier et al. (2008)
3.	*Eimeria. acervulina*	7.5 × 10^4^ oocysts/bird	Collier et al. (2008)
4.	*Clostridium perfringens*	10^7^ − 10^9^ CFU/g of intestinal contents	Shojadoost et al. (2012), Timbermont et al. (2009).

### Antibiotic growth promoters and alternatives

Generally, antibiotic growth promoters were used to improve growth performance and control of NE in poultry birds. Subsequently, over use of antibiotics developed resistance against pathogens and had hazardous effects on public health in terms of withdrawal of antibiotic residues in broiler meat (Lekshmi et al. 2017). Besides this, multidrug-resistant bacteria were isolated from broilers and were supposed to be the cause of disease in humans that led to restriction of antibiotics by EU (Castanon 2007). The antibiotic therapy destructs the normal micro flora present in GIT (Danzeisen et al. 2011; Goldstein 2011) and allows the proliferation of pathogens which irritates gastric mucosa and cause severe antibiotic-associated diarrhea (Coté and Buchman 2006). Since the ban on use of antibiotics, the incidences of NE are being increased in broiler chickens (Van Immerseel et al. 2009) and there is an urgent need to search effective alternate strategies to antibiotics. Previously, numerous investigations proved that probiotics, plants (Whelan et al. 2018), enzymes (Engberg et al. 2004), organic acids (Timbermont et al. 2010), and lysozyme (Liu et al. 2010) increase digestion and asbroption of nutrients. Among several alternative strategies, probiotics are the most effective strategy that can be used to control and prevent NE in broilers.

### Probiotics and production of short chain fatty acids (SCFAs)

**Fig. 2:**
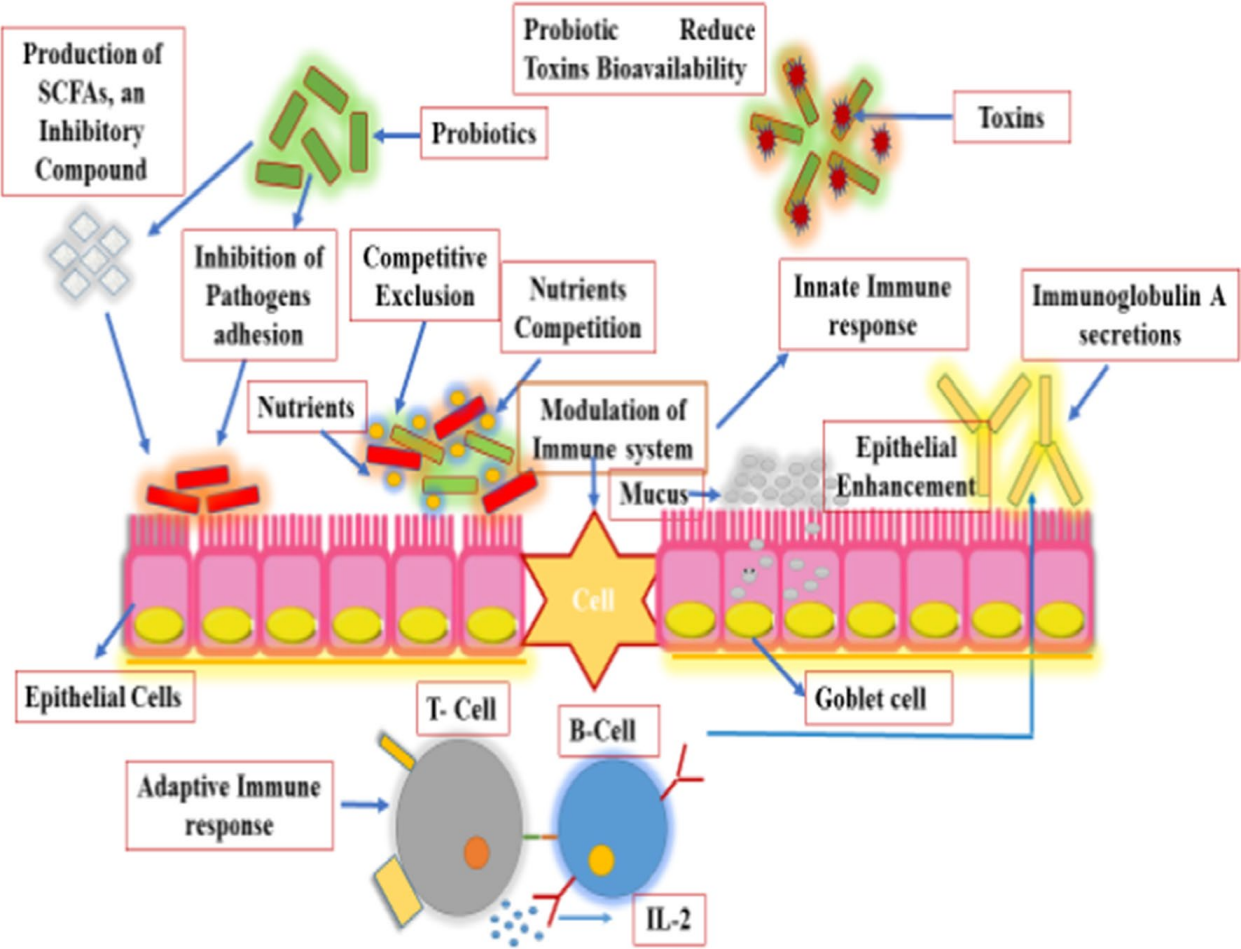
Probiotics reduce colonization of pathogens through competitive exclusion (CE) and enhance immune response

**Fig. 3:**
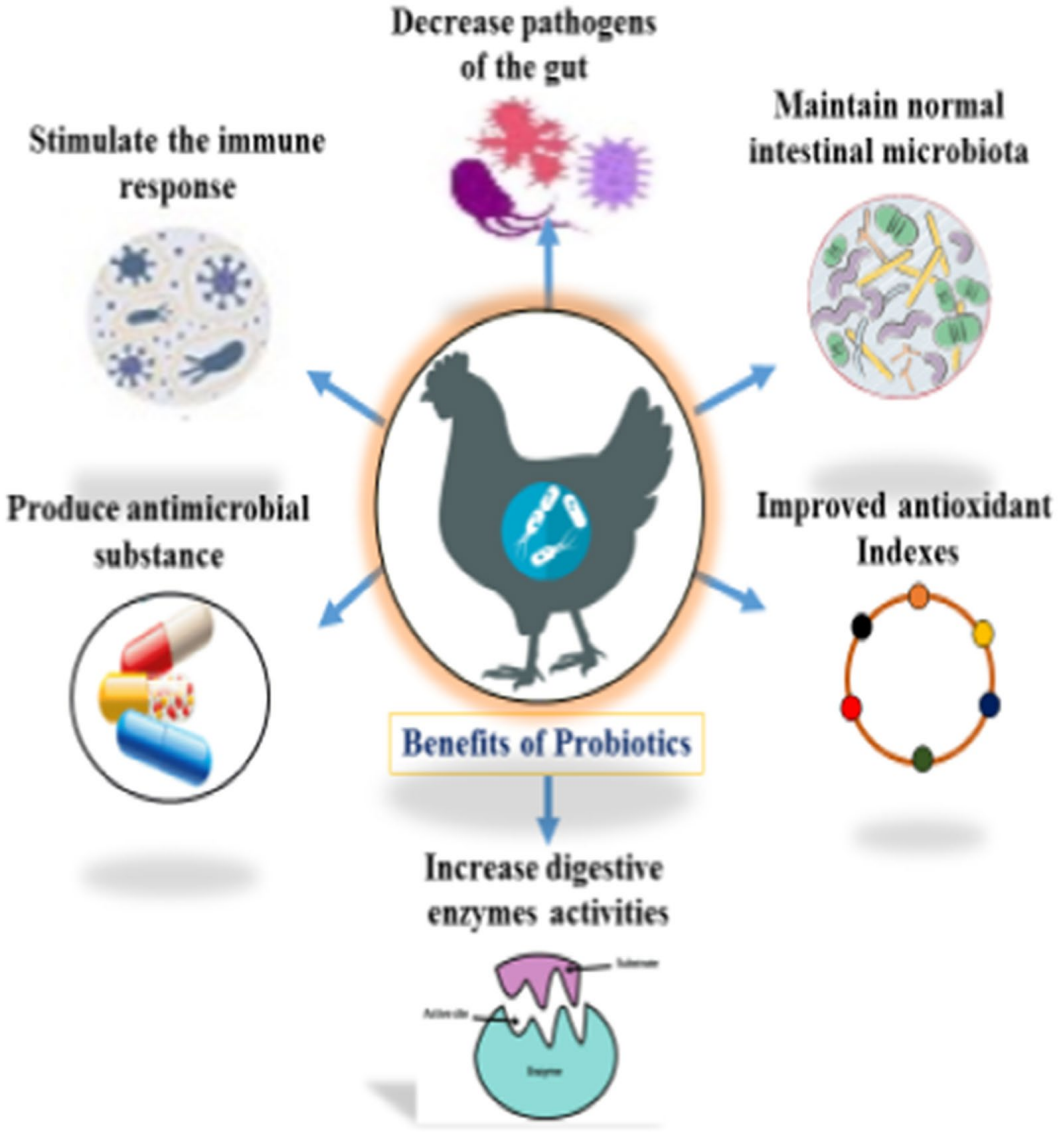
Probiotics perform many functions in broiler chickens

“Probiotics are live microorganisms, when administered in adequate amounts confer a health benefits on the host” (Fao/Who 2001; Hill et al. 2014). Probiotics or direct-fed microbial (DFM) has got great attention due to development of bacterial resistance against antibiotic and subsequently ban on AGPs in animal production (Lekshmi et al. 2017). Different strains of bio-therapeutic agents have different modes of action such as nutrients competition and adhesion with enteric pathogens, as demonstrated in (Fig. 2). Probiotic bacteria aid in digestion and absorption of nutrients by producing hydrolytic enzymes such as amylase, lipase, and protease. Also, these beneficial bacteria enhance immunity by modulating the immune system of the host and altering microbial activities in the intestine (Pourabedin et al. 2015). These live microorganisms have shown defensive characteristics by protecting intestinal mucosa which acts as a barrier as well as function as biological antagonist during clinical trials (Jones et al. 2015), probiotic bacteria perform several functions are shown in (Fig. 3). Besides, probiotics produce antimicrobial substances which inhibit the growth of pathogenic bacteria and neutralize the effects of enterotoxins (Wu et al. 2018). Another action is to stimulate the growth and development of immune organs by peptidoglycan components, present in the bacterial cell wall (Gadde et al. 2017).

SCFAs are produced by useful bacteria which are frequently absorbed and used as an energy substrate by epithelial mucosa of the intestine (Bergman 1990). Briefly, probiotic bacteria modify the gut pH by producing SCFAs which inhibit the proliferation of pathogens and consequently prevent from intestinal diseases (Jerzsele et al. 2012). In addition, supplementary SCFAs in feed stimulate the immune response (Brisbin et al. 2015; Quinteiro-Filho et al. 2015) and have shown decreased pro-inflammatory cytokine expression in challenged broiler models (Zhang et al. 2011). Butyrate also plays an important role in maintaining intestinal epithelial barrier and growth performance in broiler chickens (Al-Fataftah and Abdelqader 2014; Ritzi et al. 2014). The purpose of this section is to focus on strains of probiotic bacteria which are health promoting agents and have antagonistic properties against *C. perfringens*.

### Probiotics improve gut health and prevent NE

Many strains of probiotic bacteria such as Lactic acid bacteria, Enterococci, Bacilli, and yeast etc. have shown anti *C. perfringens* activities.

### Lactic acid bacteria

Some isloted strains of Lactic acid bacteria (LAB) have probiotic properties which are being used in animals and play extremely vital role in disease prevention (Tavakoli et al. 2017). Several natural species of LAB include *Lactobacillus, Streptococcus, Enterococcus, Carnobacterium, and Lactococcus* found in GIT of humans and animals (Vaughan et al. 2005). Useful bacterial strains having probiotic properties are well documented in (Table 2). Isolated strains belonging to *Lactobacillus sp.* are used as feed supplements to protect the broiler chickens against intestinal pathogens (Higgins et al. 2008). These isolated strains of LAB act as health-promoting agents by maintaining intestinal microbiota of poultry birds (Rajoka et al. 2017). Moreover, they have shown anti-inflammatory (Schreiber et al. 2009) and bacteriostatic properties due to secreting antimicrobial substance such as bacteriocin (Chateau et al. 1993). LAB are producing acids such as lactic acid which lowers the gut pH and prevent proliferation of *C. perfringens* found in the intestine (Liu et al. 2014). Additionally, differential abilities of *Lactobacillus *sp. as probiotic properties (*L. acidophilus*, *L. reuteri* and *L. salivarius*) are to modulate the immune response and accelerate the process of phagocytosis by macrophages in broilers (Brisbin et al. 2015). Each probiotic strain has different modes of action for inhibiting growth of pathogens and their toxic metabolites.

**Table 2:** Dose of different beneficial strains of probiotics which can be used as prophylactic remedy against necrotic enteritis in broiler chickens

Serial no.	Probiotic	Dietary dose	References
1.	*Lactobcillus acidophilus* D2/CSL	1.0 × 10^9^ CFU/kg of feed	De Cesare et al. (2020)
2.	*Lactobacillus plantrum* B1	2 × 10^9^ CFU/kg of feed	Peng et al. (2016)
3.	*Enterococcus faecium* NCIMB 11181	5.0 × 10^7^ CFU/kg of feed	Wu et al. (2019b)
4.	*Bacillus licheniformis*	1.0 × 10^6^ CFU/g of feed	Liu et al. (2017)
5.	*Bacillus subtilis*	1.0 × 10^8^ CFU/kg of feed	Rajput et al. (2013)
6.	*Saccharomyces boulardii*	1.0 × 10^8^ CFU/kg of feed	Rajput et al. (2013)

*Lactobacillus johnsonii* (LJ) BS15 is an isolated probiotic strain which can mitigate the inflammation of mitochondria, maintains gastrointestinal homeostasis, and inhibits the non-alcoholic fatty liver disease in obese mice (Xin et al. 2014). The use of *L. johnsonii* BS15 in-feed enhances growth performance and nutritional value of meat including fatty acids composition particularly the polyunsaturated fatty acid (PUFA) contents (Liu et al. 2017). Additionally, the application of *L. johnsonii* BS15 in broiler chickens can control lipid deposition and metabolism during SNE infection (Wang et al. 2017) and helps in controlling SNE (Xin et al. 2014). Other probiotic strain of *Lactobacillus plantarum* and *Lactobacillus reuteri* improve weight gain by enhancing gut health, increasing intestinal villi height, depth of crypts and decreasing intestinal viscosity (Peng et al. 2016). A solid-state fermented probiotic improves feed efficiency likely through increased energy and protein retention (Shim et al. 2010). An isolated strain of *L. plantarum* increases the proliferation of peripheral lymphocytes and expression of interferon-gamma, interleukin 6 (IL-6) and IL-10 in the intestinal mucosa. While, another probiotic strain named as *L. reuter* increases the levels of serum immunoglobulin A (IgA), IgG, and IgM in healthy chickens (Wu et al. 2019). Conclusively, the inclusion of *L. plantarum* and *L. reuteri* strains result in immediate response to disease causing pathogens such as *E. coli, C. perfringens* and *Brachyspira pilosicoli* by increasing the level of serum IgG and decreasing pathogenicity associated with intestinal infection (Ding et al. 2019).

### Enterococci

A strain of *Enterococcus faecium* (*E. faecium*) is normally found in GIT of humans and animals. The isolated strains of *enterococcus sp.* have anti-*C. perfringens* properties because they generally produce antimicrobial substances, called enterocins and organic acids (Klose et al. 2010) which help in killing pathogens present in the GIT of broilers. European Food Safety Authority (EFSA) has approved *E*. *faecium* strain 11181 as a feed supplement for improving growth performance of animals (Pajarillo et al. 2015). Moreover, a study has shown that inclusion of *E. faecium* increases the villi surface area and architecture of intestine of the host (Samli et al. 2007), help in nutrients absorption and ultimately improve weight gain. These probiotic bacteria also modulate the composition of microflora (Luo et al. 2013) and stimulates the intestinal immune response. In various investigation *E. faecium* strain has demonstrated the resistance against intestinal pathogens include Salmonella, *E. coli*, *Compylobacter* and *C. perfringens* (Karaffová et al. 2017). Thus, the inclusion of *E. faecium* in-feed can be used to control the NE incidence and health status of broiler chickens.

### Bacilli

Over the last few decades, some strains of *Bacillus sp.* such as *B. licheniformis* and *B. subtilis* have been uses as probiotics. The genus *Bacillus* has multiple advantages such as existence in the complex feed manufacturing processes, increased shelf life and robustness under fluctuating conditions within the GIT of broilers (Grant et al. 2018; Mingmongkolchai and Panbangred 2018). In poultry industry, the *Bacillus *sp. has become highly interesting due to spore-formation, which has ability to survive in harsh environment during pelleted feed processing. They are tolerant to acidic condition (low pH) and hydrolytic enzymes present in the GIT of broiler chickens (Elshaghabee et al. 2017). An investigation has shown that *Bacillus *sp*.* have anti-inflammatory properties (Eichner et al. 2018) that could prevent inflammation of intestinal mucosa.

A well known strain of *B. subtilis* as probiotic supplementation prevents proliferation of *C. perfringens* and improves body weight gain (Wu et al. 2018). Oral administration of *B. subtilis* not only modulates the intestinal health and immunity of broilers (Li et al. 2017), but also improves architecture and height of intestinal villi (Pluske et al. 1996) so that maximum absoption of nutrients could take place. Apart from this, the interaction between secretion produced by probiotic bacteria and pathogens play a key role in boosting immunity and makes intestinal structure healthy (Rajput et al. 2013). *B. subtillis* PB6, a beneficial strain isolated from the gut of healthy chickens produced an antimicrobial substance in vitro with bacteriostatic activity against various strains of *Clostridium *sp. (Elshaghabee et al. 2017), *Escherichia coli*, and *Campylobacter *sp. (Teo and Tan 2006). Most importantly, the *B. subtillis* PB6 secrets surfactins that have shown anti-microbial (Teo and Tan 2006), anti-viral and anti-tumour properties (Heerklotz and Seelig 2001) which neutralize the effects of pathogens and their toxic metabolites. Conclusively, probiotic PB6 can mitigate the NE in broiler chickens challenged with *C. perfringens *(Jayaraman et al. 2017).

Another isolated strain of *B. licheniformis*, documented as safe bacteria, is vastly used in the poultry industry with probiotic properties. Inclusion of *B. licheniformis* in-feed can be used to improve growth performance (Xu et al. 2014) as well as substitute to antibiotics for controlling NE in commercial poultry farming (Attia et al. 2012) with no adverse effects. Additionally, *B. licheniformis* produces several hydrolytic enzymes such as protease, lipase and amylase which increase the digestibility of nutrients and absorption ability in broilers (Rozs et al. 2001). In broiler chickens, the liver is a metabolic centre for lipid metabolism (Theil and Lauridsen 2007) and synthesis of fatty acids (Huang et al. 2013). Though, various stress factors influence the lipid metabolism in the commercial poultry industry (Saneyasu et al. 2013) and NE affected liver undergoes pathological changes (Yang et al. 2010). However, they are capable of reducing antioxidant stress and regulate the level of certain genes expression related to lipid metabolism. Decisively, pretreatment with *B. licheniformis* has potential to prevent poultry birds challanged with *C. perfringens* (Zhou et al. 2016).

### Saccharomyces yeast

Some strains of yeasts have shown probiotic properties belonging to the genus saccharomyces, firstly recognized by Henri boulard in 1920s from litchi fruit (Kotowska et al. 2005). Products of yeast are natural growth promoters. Oral administration of *S. boulardii* reinforces the gut ecosystem, modulates the intestinal structure, and increases production of cytokines to strengthen the intestinal mucosa against pathogenic bacteria (Rajput et al. 2013). Additionally, *S. boulardii* probiotic could improve growth performance and defense against certain pathogens (Rajput and Li 2012) by enhancing immunity of the host. Simultaneously, it enhances activities of trophic factors such as nutrients transportation (Buts et al. 1994), and shows anti-inflammatory properties (Ozkan et al. 2007). Similarly, *S. cerevisiae* also posses probiotic properties which produces antagonistic effects against pathogens in the lower part of the small intestine that may be due to the production of ethanol (Etienne-Mesmin et al. 2011). Hence, the use of yeasts strains in-feed could inhibit the growth of pathogenic bacteria and improve health status of birds as an ameliorative strategy to antibiotics.

### Role of mucosal immunity and probiotics in controlling NE

Intestinal mucosal immunity also known as gut-associated lymphoid tissue (GALT) plays a protective role against pathogens include *C. perfringens*. Generally, pathogens or foreign antigens enter in the body via penetration of mucus membrane include the epithelial lining of respiratory, gastrointestinal and urogenital tracts. However, intestinal epithelium is capable to distinguish the harmful and friendly microorganisms (Dogi et al. 2008). In GIT, beneficial bacteria protect the intestinal mucosa against pathogens where they provide a physical barrier to defend against certain pathogens (Villena et al. 2011). Moreover, the intestinal epithelial surface is covered by mucus; first barrier against admittance of infectious agents while mucosa-associated lymphoid tissue (MALT) such as adenoids and tonsils or enteric Peyer’s patches are crucial organs for the protective immune response (Johansson et al. 2011). In addition, probiotics have shown multiple positive effects in stimulating the immune response such as activation of Toll-like receptors (TLRs) that can recognize microorganism’s compound in results probiotic stimulates immune activation (Sato et al. 2009) and modulates cytokines production in broilers (Paul et al. 2013). Intestinal crypts secrete the mucus, while the function of Goblet cells is to secrete mucin and moves them between intestinal villi. Furthermore, Paneth cells are also important epithelial cells for secreting anti-microbial peptides (Johansson et al. 2011). Besides mucus and intestinal epithelial cells (IECs) are closely interlinked through tight junctions which were recorded as physical barrier to commensal bacteria as well as pathogens. Beneath this layer of epithelial cells there is lamina propria, containing the GALT that provide an immune response to the enteric mucosa. Increased numbers of lymphoid tissues and immune cells have great importance in protecting gut from the pathogens. The GALT consists of lymphoid follicles, including mesenteric lymph nodes and Payer’s patches present in small intestine (Delcenserie et al. 2008).

Conclusively, NE is one of the most important devastating intestinal diseases of poultry birds, which causes significant economic losses in terms of reduced production. Many factors negatively affect the GIT of birds and consequently impair nutrients absorption. Additionally, several factors are involved in the development of NE such as high fish meal, dysbiosis, immunosuppression and protozoal infection. However, several pharmaceutical therapies were used to control the NE in broilers but strictly banned by the EU and forced the researchers to discover suitable alternatives to antibiotics. Recent investigations have delineated that use of probiotics in poultry feed stimulates the immunity of the host through antimicrobial substance called bacteriocins which not only inhibit the growth of *C. perfringens* but also improve gastrointestinal health of the birds. Thus, the use of probiotics in feed supplement also improves weight gain through improving intestinal villi height which helps in absorption of the nutrients. Moreover, the use of these beneficial probiotics can increase the profitability, poultry production and produce good quality meat for consumers. Probiotic bacteria are recognized as one of the most suitable alternative strategies to antibiotics that could reduce the hazardous effects of antibiotics. In future, there is a need to isolate more strains of useful bacteria having probiotic properties that could be used instead of antibiotics for controlling NE in broilers.

## Data Availability

Not applicable.
